# Optimization of bacterial colonization in the gut of axenic and conventional zebrafish larvae using live food

**DOI:** 10.1128/spectrum.02853-25

**Published:** 2026-04-21

**Authors:** Gabriel Byatt, Cédric Goffard, Jeffrey K. Cornuault, Mado Lemieux, Paul De Koninck, Marie-Eve Paquet, Sylvain Moineau

**Affiliations:** 1 Département de biochimie, de microbiologie et de bio-informatique, Faculté des sciences et de génie, Université Laval 4440, Québec City, Quebec, Canada; 2 CERVO Brain Research Centre 33499 , Québec City, Quebec, Canada; 3 Department of Microbiology and Immunology, McGill University 5620, Montreal, Canada; 4 Département d’anesthésiologie et soins intensifs, Faculté de médecine, Université Laval 4440, Québec City, Quebec, Canada; 5 Félix d’Hérelle Reference Center for Bacterial Viruses, Université Laval 4440, Québec City, Quebec, Canada; Anhui Medical University, Hefei, China

**Keywords:** zebrafish, larvae, *Tetrahymena*, bacterial colonization, fluorescence, gut microbiota, *E. coli*

## Abstract

The zebrafish increasingly serves as a model for studying the influence of the gut microbiota on the physiology of the host. In an experimental setting, specific bacteria can be used to colonize the gut of zebrafish at an early stage (larvae), affecting their development, physiology, behavior, and overall health. However, the efficiency of gut microbial colonization protocols in zebrafish larvae depends on several factors, such as bacterial strains, zebrafish lines, pre-colonization status, timing of colonization, the type of food vector, and assay methods. In this study, we tested how different parameters influence bacterial colonization in the gut larvae from various genetic lines, both axenic and conventional. To demonstrate gut colonization in transparent larvae up to 9 days post-fertilization (dpf), we used an *Escherichia coli* strain expressing fluorescent proteins and fluorescence microscopy. Additionally, we utilized plate counts to quantify the extent of bacterial colonization. We found that introducing live food (*Tetrahymena thermophila*) loaded with bacterial cells strongly facilitated colonization, increasing the initial bacterial load by one log. We also examined the impact of larval density and timing of exposure to bacteria, which together enabled successful bacterial colonization of the zebrafish larvae’s gut with increasing bacterial cell counts (from 10^4^ to 10^6^) over four days. We also successfully colonized the zebrafish larvae with strains of *Pseudomonas aeruginosa*, *Aeromonas veronii*, and *Vibrio cholerae*. Implementing standardized and optimized bacterial colonization protocols in zebrafish larvae should facilitate the use of this model to study the interactions between the gut microbiota and host physiology.

**IMPORTANCE:**

Zebrafish larvae hold potential as a model for studying gut microbiota. However, standardized protocols for controlling bacterial colonization of the larval zebrafish gut are required. This article presents the analysis of various parameters that led to an effective method for colonizing zebrafish larvae with various bacteria. The proposed detailed protocol enables the qualitative, quantitative, and reproducible observation of bacterial colonization in the zebrafish larvae’s gut.

## INTRODUCTION

To gain insights into how the gut microbiota influences host health, employing an animal model that allows for precise and reproducible manipulation of its microbial content is desirable. Over the last decade, the zebrafish has emerged as a model for investigating gut microbiota, thanks to its amenability for high-throughput investigations, availability of multiple mutant and transgenic lines, and optical transparency (1, 2). Two decades ago, Rawls et al. pioneered the use of a synthetic microbiota to colonize germ-free zebrafish larvae, which was achieved by immersing them in a medium containing 10^2^ to 10^4^ bacteria per milliliter (3, 4). Using 16S sequencing, they detected various bacterial genera and species in the larval gut. Their work served as a blueprint for subsequent studies employing PCR-based bacterial detection following colonization in the zebrafish gut (5, 6). While these sequencing-based approaches offer advantages, they also have limitations, such as susceptibility to false positives stemming from the detection of free DNA or dead bacteria and the inability to longitudinally monitor colonization in the same larvae (7).

By leveraging the transparency of the zebrafish during its larval stage and the use of fluorescently labeled bacteria, we can track viable colonizers in real time and longitudinally (8–10). In most studies using fluorescent bacteria to track colonization in zebrafish, microscopy observations were usually limited to the first 48 h after bacterial exposure, either ending once the fluorescence signal disappeared or stopping without further monitoring, which corresponds to the typical time required for gut content to be expelled (8, 11, 12). This short observation window may not be sufficient to distinguish gut colonization from transient passage of bacteria (13). In addition, the fluorescence intensity can be difficult to assess, depending on the fluorescent marker used, the size and positioning of the larvae under the microscope, and the heterogeneity of the localization of the fluorescent signal in the gut (14, 15). Combining both fluorescence and PCR readouts can help mitigate these limitations. Furthermore, bacterial counts (colony-forming units, CFU) can offer a more precise evaluation of the quantity of viable bacteria within the gut (3, 16, 17). While there is a wide availability of transgenic and mutant lines of zebrafish, it remains to be tested whether their gut bacterial colonizing potential is robust.

Zebrafish larvae are typically maintained in a specialized liquid medium, which usually has a low nutrient concentration that does not support bacterial growth nor a healthy gut microbiota in the absence of feeding (18). The zebrafish gut typically becomes fully functional around 5 days post-fertilization (dpf), coinciding with the onset of feeding (19). The process of food digestion typically spans from 6 to 24 h (13). Adequate nutrient availability is vital not only for fish development but also for promoting bacterial colonization and growth (20). Zebrafish larvae can be fed with dry and/or live food, with the latter offering the advantage that zebrafish exhibit a preference for moving prey. Live food enhances their predatory behavior and overall food intake. A study showed that introducing bacteria into the zebrafish intestinal tract is more effective when these bacteria are initially incorporated into a food vector, such as a ciliate, as opposed to adding bacteria to fish tank water (21). *Tetrahymena thermophila* is a ciliate candidate as a food source in the context of bacterial colonization due to its mobility and ability to internalize live bacteria (22, 23). Yet, several factors may affect the quantity of bacteria consumed by zebrafish larvae, which can lead to variability in colonization efficiency.

Numerous studies have harnessed the zebrafish model to manipulate gut microbial colonization and explore its roles in various biological responses and disorders (6, 12, 21, 24–26). In the adult zebrafish gut microbiota, the predominant bacterial phyla are *Bacillota* (*Firmicutes)* and *Proteobacteria*, while in the mammalian gut, *Bacillota* and *Bacteroidota* (*Bacteroidetes*) dominate (27, 28). Although *Bacteroidota* are present in zebrafish, they are in relatively low abundance. Bacteria known to be present during the early stage of development of the larval gut include *Vibrio cholerae*, *Aeromonas veronii*, and *Pseudomonas aeruginosa (*3, 16, 28). To establish a robust colonization protocol, we employed *Escherichia coli,* a species commonly selected for model development due to the wealth of knowledge and the wide array of tools available (plasmids, protocols, etc.). While not typically part of the zebrafish microbiota, this species is able to survive in its gut, and gammaproteobacteria have been observed in this environment (4, 16, 17, 29). It is also commonly found in the mammalian gut, including in early stages of life (30, 31).

In this study, we tested several parameters to enhance bacterial colonization in the zebrafish larvae gut, including the addition of *T*. *thermophila* as live food and as a vehicle for bacteria delivery, the ratio of available food per larva, and the timing of exposure. The optimized parameters were tested in both axenic and conventional zebrafish larvae, from different zebrafish lines, and using various fluorescent bacterial strains. The axenic condition was first utilized to eliminate the influence of endemic bacteria on gut colonization by new bacteria. We then tested the established protocol for the introduction of specific bacteria in conventionally raised larval zebrafish.

## MATERIALS AND METHODS

### Zebrafish husbandry

Zebrafish were maintained, bred, and manipulated at the Laboratoire aquatique de recherches en sciences environnementales et médicales (LARSEM) of Université Laval. Embryos produced for the experiments were transferred to the CERVO Brain Research Centre, where all the assays were performed. All manipulations and procedures on adults and larval zebrafish followed strict protocols approved by the animal care committee of Université Laval. Four zebrafish lines [tg(APOEb:lyn-GFP), WT-AB, WT-TL, and Casper WT (32–34)] were maintained in a Techniplast’s ZebTec zebrafish housing system with 90% water recirculation at 28.5°C ± 0.5°C and light/dark cycles of 14/10 h. Fish were monitored daily by trained technicians. Adult zebrafish were fed dry (Gemma Micro) and live (Artemia) food (35). Fish were bred at most once a week and eggs were placed in a sterile Petri dish or in a multi-well plate and incubated at 28.5°C with light/dark cycles of 14/10 h. Larvae were kept for up to 10 dpf in embryo medium (EM) (13.7 mM NaCl, 0.54 mM KCl, 1.0 mM MgSO_4_, 1.3 mM CaCl_2_, 0.025 mM Na_2_HPO_4_, 0.044 mM KH_2_PO_4_, 4.2 mM NaHCO_3_, and adjusted to pH 7.2 with 12 N HCl). The larvae were fed with *T. thermophila,* and EM was refreshed daily. During the colonization assays, zebrafish larvae were maintained in 6-well plates containing 5 mL of EM and up to five larvae/well. After 10 dpf, zebrafish larvae were euthanized by combining rapid cooling and an overdose (1.15 mM) of tricaine methanesulfonate (TMS). WT-TL and WT-AB zebrafish lines were treated with phenylthiourea (PTU, 0.2 mM) to slow the pigmentation formation starting at 24 h post-fertilization.

### *Tetrahymena* culture

*T. thermophila* CU428.2 (Cornell University) was grown in SPP medium (20 g/L proteose peptone, 10 mM dextrose, 1 g/L yeast extract, 0.033 mM FeCl_3_) at 28.5°C, and fresh cultures were prepared every 3–4 days. *Tetrahymena* were received axenic and kept axenic in SPP medium containing antibiotics (streptomycin 430 uM, penicillin 748 uM, amphotericin B 0.27 uM). Ciliates were centrifuged (2 min, 600 × *g*) and washed three times with sterile EM. *T. thermophila* was added to the larvae medium to obtain a final concentration of 10^4^/mL. Fresh *Tetrahymena* were added daily when the EM was changed. For the pre-fed condition, *Tetrahymena* were washed three times in sterile EM and then incubated with washed bacteria for 1 h, in a ratio of 1:100 ciliate-bacteria. Ciliate engorgements with bacteria were observed with an inverted epifluorescence microscope (Zeiss AXIO Observer.Z1).

### Bacteria

Strains used in this study are listed in Table 1. *E. coli* was incubated at 28.5°C in LB medium with ampicillin (100 μg/mL), arabinose (2 mg/mL), and agitation at 250 rpm. Each *E. coli* strain was transformed by electroporation with a plasmid harboring a gene coding for the red fluorescent protein mCherry2 under an arabinose-inducible promoter (pBAD/HIS-B mCherry2). After 16 h of incubation at 28.5°C with agitation, an OD_600nm_ of 1.0 was obtained (~1 × 10^9^ CFU/mL). Cells were washed three times in EM by centrifugation (3,000 × *g* for 2 min), after which the supernatant was removed and replaced with EM.

**TABLE 1:** Bacterial strains and plasmids used in this study^*a*^

Bacterial strain, plasmid, or primer	Characteristics	Sources
*E. coli*		
MG1655	Laboratory strain	FHRCBV
NEB10	Laboratory strain	NEB
SMQ-1532	MG1655 containing plasmid pBAD/HIS-B mCherry2	This study
SMQ-1533	NEB10 containing plasmid pBAD/HIS-B mCherry2	This study
SMQ-1534	Isolated from a fecal sample of an infant (JC08, 06F05)	(36)
SMQ-1535	SMQ-1534 containing plasmid pBAD/HIS-B mCherry2	This study
SMQ-1683	SMQ-1534 containing plasmid pBAD/HIS-B mNeonGreen	This study
SMQ-1684	SMQ-1534 containing plasmid pBAD/HIS-B mCerulean3	This study
*Aeromonas veronii*		
AH-42	HER1216	FHRCBV
SMQ-1536	AH-42 containing plasmid pUCP30T-mCherry2	This study
*Pseudomonas aeruginosa*		
PAO1	HER1153	FHRCBV
SMQ-1537	PAO1 containing plasmid pUCP30T-mCherry2	This study
*Vibrio cholerae*		
Makassar 757	HER1052	FHRCBV
SMQ-1538	Makassar 757 containing plasmid pUCP30T-mCherry2	This study
Plasmids		
pBAD/HIS-B mCherry2	pBAD/HIS-B backbone with a gene coding for mCherry2 and controlled by an arabinose-induced promoter	Tokyo
pBAD/HIS-B mNeonGreen	pBAD/HIS-B backbone with a gene coding for mNeonGreen and controlled by an arabinose-induced promoter	Tokyo
pBAD/HIS-B mCerulean3	pBAD/HIS-B backbone with a gene coding for mCerulean3 and controlled by an arabinose-induced promoter	Tokyo
pUCP30T-E2crimson	Plasmid used for cloning the pUCP30T-mCherry2 plasmid	(35)
pUCP30T-mCherry2	pUCP30T with a gene coding for mCherry2 fluorescent protein and controlled by an IPTG-induced promoter	This study
Primers (5′–3′)		
JC142	GCCGATGATGGGGATCCACTAGTCGCCACCATGGTGAGCAAGGGCGAGGA	This study
JC143	GTCGTGCTTGTACAATTCGTCCATACCGTGTTACTTGTACAGCTCGTCCATGCCG	This study
JC145	CACGGTATGGACGAATTGTA	This study
JC146	GGTGGCGACTAGTGGATC	This study
16S amplicon PCR forward primer	TCGTCGGCAGCGTCAGATGTGTATAAGAGACAGCCTACGGGNGGCWGCAG	(37)
16S amplicon PCR reverse primer	GTCTCGTGGGCTCGGAGATGTGTATAAGAGACAGGACTACHVGGGTATCTAATCC	(37)

^*a*^ FHRCBV, Félix d’Hérelle Reference Center for Bacterial Viruses, https://www.phage.ulaval.ca/; NEB, New England Biolabs; University of Tokyo, gift from Robert E. Campbell.

*P. aeruginosa* PA01, *A. veronii* AH-42, and *V. cholerae* Biotype El Tor Makassar 757 (Table 1) were individually transformed by electroporation with the broad-range vector pUCP30T-mCherry2. This plasmid was built from pUCP30T-E2crimson (Addgene plasmid #78478 [38]), in which the gene coding for E2crimson was swapped with the one coding for mCherry2 (Table 1). The expression of this protein was induced by isopropyl β-D-1-thiogalactopyranoside (IPTG) (0.05 mM). The three bacterial strains were cultivated overnight at 28.5°C in LB with agitation (250 rpm).

### Procedure to obtain axenic zebrafish larvae

Protocol was inspired by Melancon et al. (39). One hour after fertilization, eggs were washed three times with sterile EM. Clean eggs were transferred into sterile EM containing antifungal and antibiotics (EM + antibiotics: amphotericin B at 8.4 µM, ampicillin at 300 μM, and kanamycin at 200 µM). After 90 min of incubation at 28.5°C, the medium was replaced with fresh EM + antibiotics. Two hours after the second incubation, eggs were washed three times with sterile EM and incubated in a 0.2 μm filtered povidone-iodine (100 g/L) solution for 2 min, washed three times with sterile EM, and incubated in filtered sodium hypochlorite (0.4 mM) for 20 min. Finally, sterilized eggs were washed three more times with sterile EM and were transferred to sterile T75 cell culture flasks. Axenicity was tested by plating 500 uL of media on TSA and LB plates (with no antibiotics) after every treatment and by PCR targeting the 16S rRNA gene. Primers used for the PCR are listed in Table 1. The cycle used was as follows: denaturation for 2 min at 95°C, 30 cycles of denaturation at 95°C (30 s), hybridization at 55°C (30 s), and elongation at 72°C (2 min). These cycles were followed by a final step of 5 min at 72°C.

### Bacterial colonization of zebrafish larvae

Zebrafish were dechorionated at 2 dpf by pipetting up and down the eggs in EM with a long glass Pasteur pipette. Overnight bacterial cultures were washed with sterile EM and resuspended at a final concentration of 10^6^ CFU/mL in wells containing 2 dpf zebrafish larvae. This bacterial concentration was shown previously to have minimal impact on fish survival while favoring colonization of zebrafish larvae (17). The larvae were incubated with bacteria and *Tetrahymena* from 2 to 5 dpf at 28.5°C (3). After the incubation period (5 dpf), the EM was replaced, and the larvae were transferred into a sterile 6-well plate. In the case of the experiment on the onset of exposure, bacteria were added at 5 dpf instead of 2 dpf. Following this step, only *Tetrahymena*, arabinose, ampicillin, and phenylthiourea (PTU, 0.2 mM) were added to the wells. Larvae were transferred into fresh sterile EM every 24 h. Movie S1 shows live and moving *Tetrahymena* engorged with fluorescent *E. coli*. For colonization of conventionally raised zebrafish larvae, we exposed the larvae to pre-fed *Tetrahymena* from 5 to 6 dpf. Larvae were then replaced into a sterile 6-well plate and kept in EM with arabinose. Every experiment with colonization was done in triplicate, with three different hatchings.

### Imaging fluorescent bacteria in the gastrointestinal tract of zebrafish larvae

The larvae were individually picked from the EM, washed, embedded in 2% agarose in 24-well plates, and set aside for fluorescent microscopy. Images were acquired with the ZenPro software package (version 3.7) on an inverted epifluorescence microscope (Zeiss AXIO Observer.Z1) equipped with a light source (12 V 100 W Halogen Lamp, Lumencor Spectra7 Led), 5× magnification objective (Zeiss, 0.15 Ph1), and using the Zeiss filter set 00 for mCherry2 or the Zeiss filter set 38 HE for GFP. An exposure time of 300 ms was used. Images were then analyzed using the image processing package Fiji from the ImageJ software. Fluorescence intensity levels were standardized across images for comparison. The area of interest was drawn around the gut or around the section of gut, and the mean fluorescence signal was calculated. Fluorescence means were represented by arbitrary units. Arbitrary units (AU) were used as units for fluorescence intensity. Spectral unmixing was done using ImageJ during the simultaneous imaging of three fluorescent proteins.

### Bacterial counts

Bacterial counts were determined on larvae at 0, 48, and 72 h after the removal of bacteria from the media. Each zebrafish larva was washed by allowing it to swim freely for 5 min in sterile EM and then euthanized with TMS (300 mg/L). Individuals were placed in 100 μL of sterile EM and homogenized for 3 × 10 s using a motorized pellet pestle. The supernatant was subjected to serial dilution in sterile EM ranging from 10^−1^ to 10^−6^, and 100 μL of the various dilutions were plated in triplicate on LB agar plates supplemented with ampicillin (100 μg/mL) and arabinose (2 mg/mL). Plates were incubated overnight at 28.5°C. Colonies were manually counted by detecting red fluorescence through Chroma autofluorescent plastic slides (Chroma, 92001), allowing a 610 nm light to pass through. Each plating was performed in triplicate as technical replicates.

### Statistics

Statistical analyses were performed using the GraphPad Prism 10.4.0 software package. Every experiment was performed using three independent groups of zebrafish larvae. Normality was first assessed with the Shapiro-Wilk test. For comparisons between two groups, either a *t*-test (parametric) or a Mann-Whitney test (non-parametric) was applied. For comparisons involving more than two groups, a one-way ANOVA, followed by a Tukey’s post hoc test, was used, or a Kruskal-Wallis, followed by a Dunn’s post hoc test. A Brown-Forsythe test was performed to evaluate differences in variance between conditions.

## RESULTS AND DISCUSSION

The main objective of this study was to test various parameters to establish a reliable protocol for efficiently colonizing the gut of zebrafish larvae with bacteria and observe subsequent bacterial growth. The general protocol begins with the sterilization of the eggshells to generate axenic larvae (39). Subsequently, the axenic hatched larvae were exposed to ciliates (*T. thermophila*) along with fluorescence-expressing bacteria and were maintained until 9 days post-fertilization, while bacterial colonization was monitored with an epifluorescence microscope, enabling non-invasive longitudinal fluorescence imaging of many zebrafish larvae. Bacterial colonization was also quantified through cell counts. Using this general protocol, we tested various conditions to optimize gut colonization.

### Exploiting a live bacterial carrier as food for zebrafish larvae

Feeding of zebrafish larvae can begin around 5 dpf, when their gut is sufficiently mature (19). A typical diet for zebrafish consists of live and moving zooplankton and unicellular organisms, which induce a predatory response, thereby stimulating feeding (40, 41). *Tetrahymena*, a motile unicellular eukaryote measuring approximately 50 μm, is known for ingesting and subsequently releasing undigested bacteria as fecal bodies (22, 42). Thus, these ciliates can act as both bacterial carriers and food for zebrafish larvae (21). *Tetrahymena* typically captures bacteria and encapsulates them in a food vacuole, where the bacterial cells can remain alive for up to 90 min (Movie S1), but also can cohabit during multiple days and can be expelled with fecal pellets (43, 44). The larval digestive process also facilitates the release of live bacteria into the lumen by disintegrating the *Tetrahymena* and allowing the release of various nutrients, which can be used by the bacteria in the gut. Previous studies have used *Tetrahymena* as a food source, while in other studies, paramecia were used as a colonization vector (29, 39, 45).

**Fig 1:**
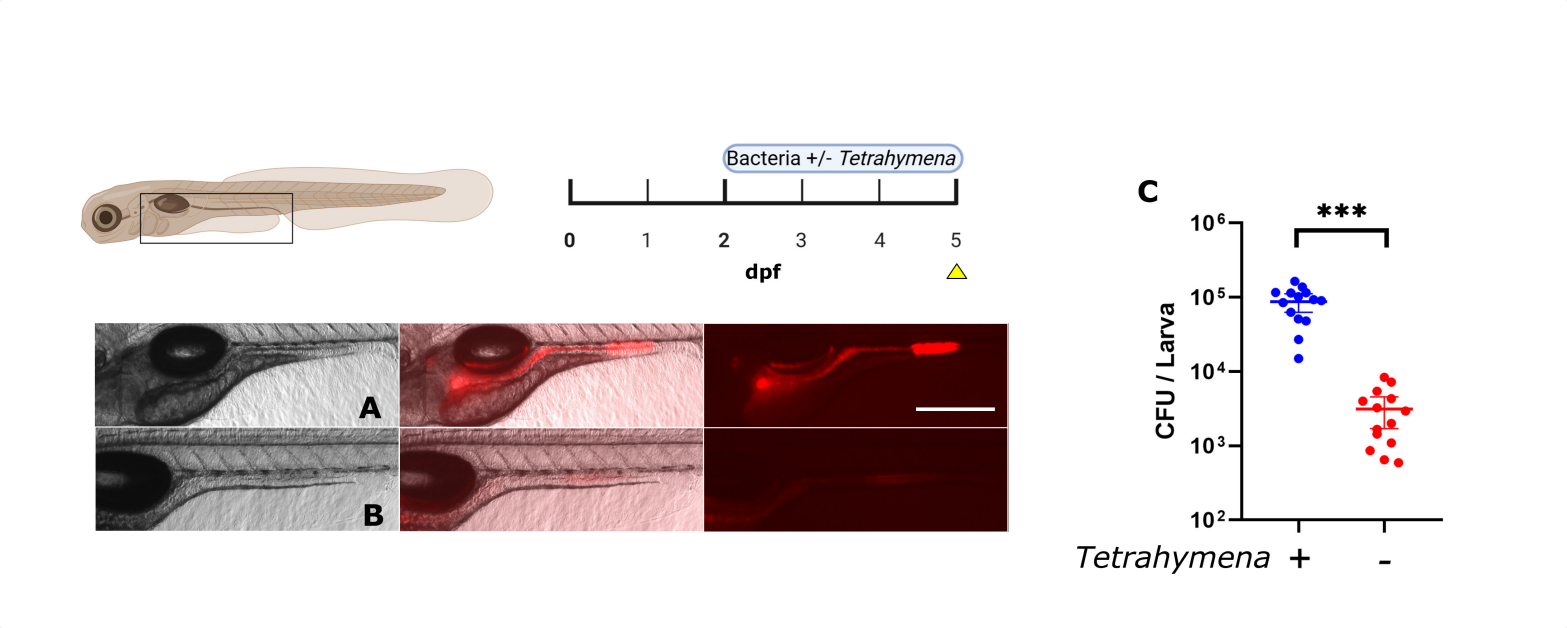
Effect of *T. thermophila* co-incubation on *E. coli* colonization. Lateral view of an axenic zebrafish larva at 5 dpf exposed to a strain of *E. coli* expressing mCherry2. Left = brightfield image, middle = merge, right = fluorescence. Every figure follows this pattern. (**A**) Larvae were exposed at 2 dpf to *E. coli* and *Tetrahymena* simultaneously. (**B**) Larvae were exposed only to *E. coli.* (**C**) Bacterial counts (CFU) per larva at 5 dpf. Scale bar: 300 µm. *N* = 14 for each condition. *t*-test was used to compare the means (*** = *P* < 0.0001). Yellow arrowhead on pictogram = moment of optical and biological observations.

We tested the effectiveness of *Tetrahymena* as a bacterial carrier for bacterial colonization of the larval zebrafish gut at 2 dpf by co-incubating *E. coli* cells expressing mCherry2 in the presence or absence of the ciliate for 3 days. Figure 1A and B show that when *Tetrahymena* were co-incubated with fluorescent bacteria, fluorescence in the larval gut (at 5 dpf) could be observed, indicating the presence of bacteria in the gut. In the presence of *Tetrahymena*, the mCherry2 signal was detected throughout the gut, mainly in the distal section, while the absence of *Tetrahymena* led to no fluorescence signals above background in the same regions. This observation was corroborated by the quantitative bacterial count, revealing a 100-fold difference between the two conditions (Fig. 1C, 8.7 ± 1.1 × 10^5^ CFU/larva with *Tetrahymena*; 3.1 ± 0.6 × 10^3^ CFU/larva without *Tetrahymena*.

### Onset of bacterial exposure

**Fig 2:**
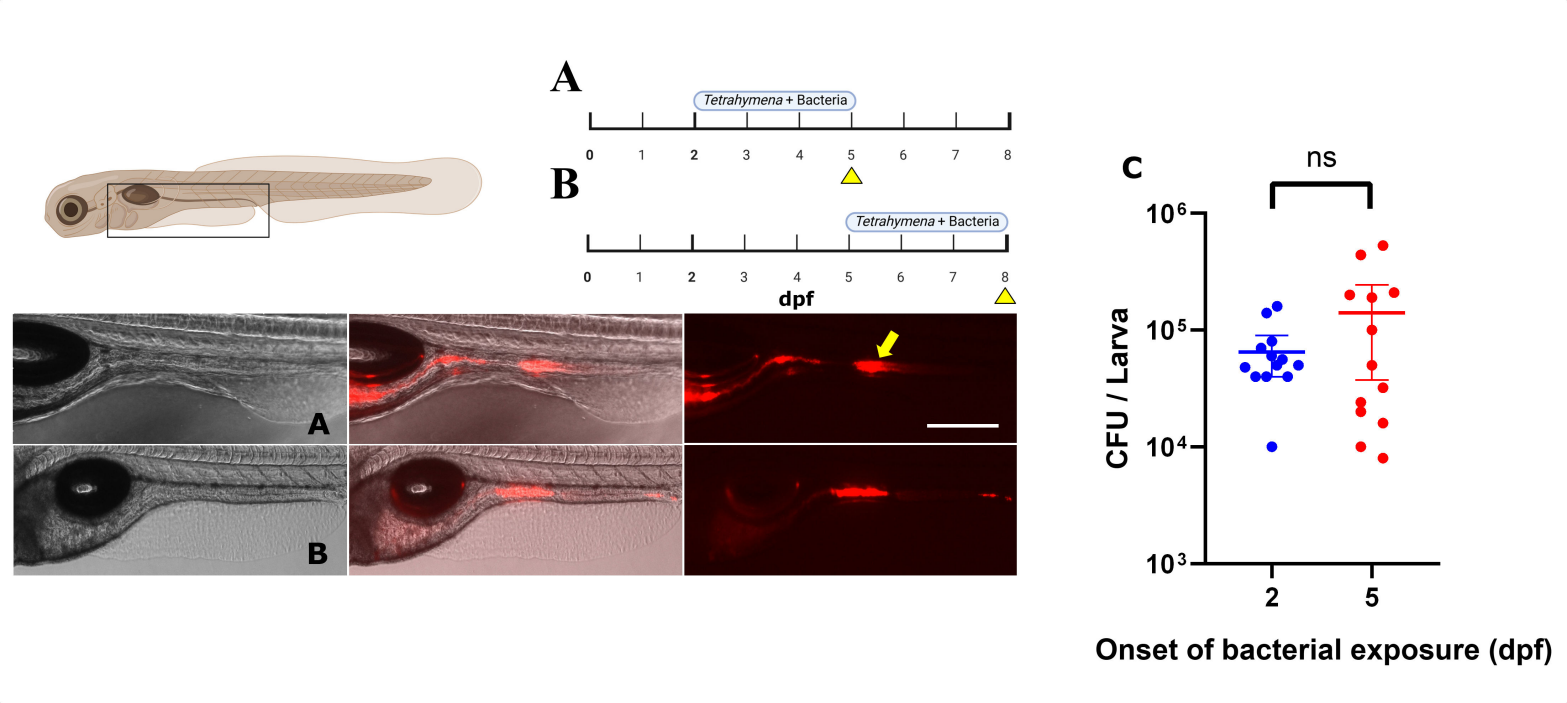
Age of the zebrafish larvae at the time of exposure to bacteria**.** Lateral view of an axenic zebrafish larva exposed for 3 days to a strain of *E. coli* expressing mCherry2. (**A**) *E. coli* cells were added at 2 dpf. (**B**) *E. coli* cells were added at 5 dpf. (**C**) Quantification of *E. coli* after exposure at 2 and 5 dpf (3 days post-exposure to bacteria [dpe] in both conditions) (CFU/larva). Scale bar: 300 µm. Both conditions fed with *T. thermophila*, *N* = between 13 and 15. *t-*test was used to compare the means (*P* = 0.1338, ns = non significative). Yellow arrow points the distal gut region (LRE).

In their natural environment, zebrafish larvae encounter microorganisms immediately upon hatching, and early gut colonization has been shown to positively impact their health (19, 25, 46). In the experiment above, bacteria were introduced at an early developmental stage (2 dpf) with *T. thermophila*. We then asked whether delaying the introduction of bacteria at 5 dpf, corresponding to the moment when the gut is fully functional, would impact colonization (Fig. 2) (19, 47). Little difference in bacterial concentration was observed between the two conditions, 3 days later (Fig. 2C; 2 dpf: 0.6 ± 0.1 × 10^5^ CFU/larva; 5 dpf: 1.4 ± 0.4 × 10^5^ CFU/larva), albeit with a broader variability (*P* < 0.0001) at 5 dpf. The larvae exposed at 2 dpf were imaged at 5 dpf, while the larvae exposed at 5 dpf were imaged at 8 dpf. In both conditions, we observed uneven fluorescence signals in the midgut section (Fig. 2A and B). This irregular distribution of fluorescence may suggest preferable sites of colonization in the zebrafish larvae’s gut by the *E. coli* strain used in this study, as described for other bacteria in the human gut (48). When quantifying the fluorescence, we observed a higher fluorescence signal in the digestive bulb and in the early mid-gut in zebrafish exposed at 2 dpf (489.3 ± 113.9 AU) compared to the fluorescence signal of the same zone of zebrafish exposed at 5 dpf (203.7 ± 55.9 AU). Bacteria can also cluster in the gut during colonization, which can lead to irregular colonization and signal in the lumen (49). We imaged the colonization every 15 min for a day and observed clusters of cells forming over time in different parts of the gut (Movie S2). However, after observing 15 larvae, we did not detect a specific region of fluorescence enrichment except for the lysosome-rich enterocytes (LRE) region (see arrow in Fig. 2A). It has been demonstrated that mCherry proteins can also be absorbed by LRE and retain their fluorescence for several hours before undergoing degradation (50). Another interpretation for the irregular distribution of fluorescence along the gut may be due to transient passage. It has been shown that food transits in the gut of larval zebrafish between 7 and 24 h, sometimes leaving some fluorescent debris in the gut lumen (11, 51).

**Fig 3:**
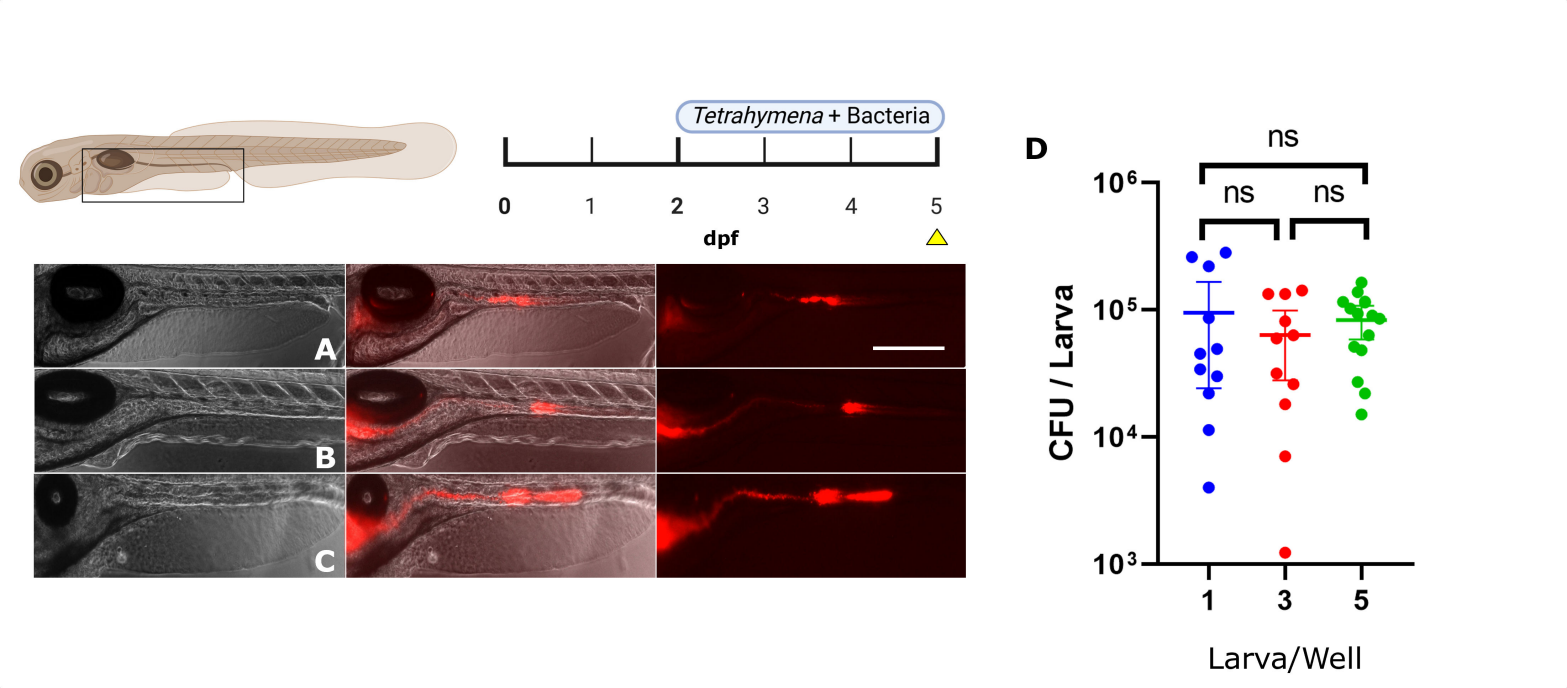
Impact of zebrafish larvae/ciliate ratio in bacterial colonization well. Red fluorescence emitted by *E. coli* cells expressing mCherry2 in colonized larvae. (**A**) One zebrafish larva per well (5 mL). (**B**) Three zebrafish larvae per well (5 mL). (**C**) Five zebrafish larvae per well (5 mL). (**D**) Number of CFU per larva for every condition. Scale bar: 400 µm. *N* = between 11 and 15 for each condition. Statistical tests: Kruskal-Wallis followed by a Dunn’s comparisons test (ns = *P* > 0.9).

Building on the findings that the addition of *Tetrahymena* enhances bacterial colonization, we tested different ratios of ciliates per larva. We used the same concentration of *Tetrahymena* and bacteria, while changing the number of zebrafish larvae per well. The population sizes tested were 1, 3, and 5 larvae, corresponding to a fivefold range in the number of available *Tetrahymena* (5 × 10^4^, 1.7 × 10^4^*,* or 1 × 10^4^ *T. thermophila* per larva in 5 mL). The resulting mean bacterial counts were not significantly different in the gut of 5 dpf larvae across all groups (Fig. 3). No significant difference in variance was observed between the conditions 1 × 10^4^ and 1.7 × 10^4^ of *T. thermophila* per larva in 5 mL. When quantified, the mean fluorescence intensity was not significantly different between the three conditions (300.4 ± 42.9 AU for 1 × 10^4^, 283.3 ± 57.5 AU for 1.7 × 10^4^, and 295.1 ± 26.1 AU for 5 × 10^4^). Larval zebrafish eat up to 10% of their weight in food daily, which represents 0.1 mg of food making. It is possible that the lowest concentration of *Tetrahymena* used in this study is higher than the normal consumption of *Tetrahymena* per day per larva, possibly explaining the similar bacterial counts (50, 52).

The benefits of employing a higher number of larvae per well become most apparent from a long-term perspective, facilitating a higher frequency of bacterial passage through the gut. This advantage may be attributed to the potential migration of bacteria from the gut to the external environment and back to the larvae (2, 53). The gut serves as a bacterial reservoir, offering time for adaptation and providing a distinct environment from the embryo media. Since *E. coli* does not thrive but survives in the EM, the larval gut may provide an environment for bacterial growth during extended exposure, consequently facilitating their migration to other larvae. It is hypothesized that each larva will consistently ingest a similar number of ciliates, resulting in a stable average bacterial count in the gut (54).

### Increase gut bacterial populations over time

**Fig 4:**
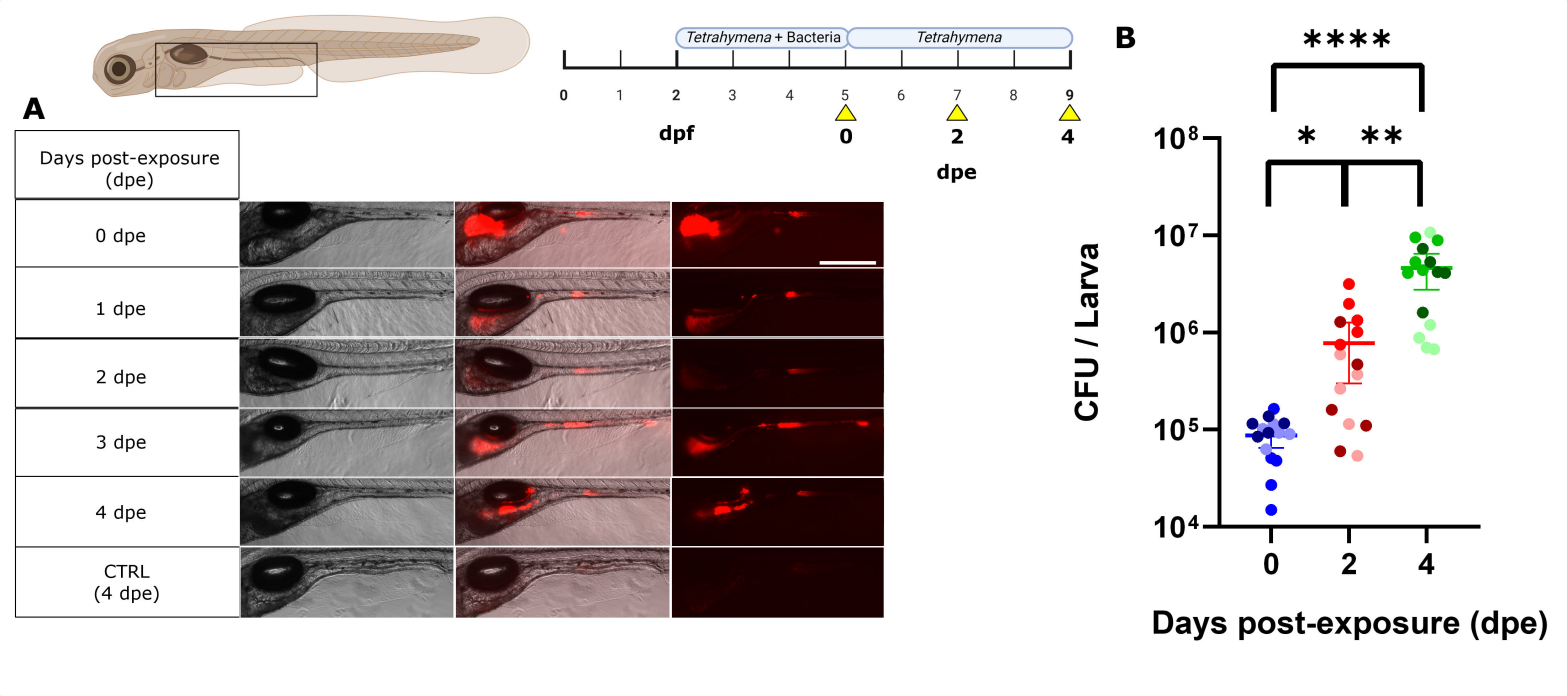
Levels of *E. coli* expressing mCherry2 in the gut of zebrafish larvae (WT-TL) over time. (**A**) Lateral view of a zebrafish larvae after the colonization protocol. (**B**) CFU counts were made at 0-, 2-, and 4-days post-exposure to media containing bacteria. Scale bar: 300 µm. *N* = 15 for each condition, from three separate experimental groups of 5, each group of 5 coming from a different hatching (*N* = 3). Each larva from a given group has the same shade of color to reflect the reproducibility of the protocol. Statistical tests: Kruskal-Wallis and Dunn’s multiple comparison test (*P* value: * = 0.0329, ** = 0.0098, **** = *P* < 0.0001).

By implementing the optimized parameters outlined in the previous sections (exposure at 2 dpf, addition of live food, ratio of ciliate per larva), we examined whether *E. coli* colonized and increased in number in the initially axenic zebrafish larvae gastrointestinal tract by removing the bacteria from the EM beyond 5 dpf. If the bacterial cells are actively colonizing rather than simply passing through, one would anticipate an increase in cell counts over time or a stable number, compensating for cell death and gut clearance. By changing the medium daily with sterile media, we ensured that the concentration of bacteria in the water remained low. We observed a progressive rise in bacterial cell counts within the gut over time after day 5, as evidenced by both fluorescence signals in the gut and CFU counts over the course of 4 days (Fig. 4). A concentration of 8.8 ± 1.0 × 10^4^ CFU per larva was initially observed at day 0 post-exposure to bacteria (dpe). Here, dpe is defined as the number of days after the final exposure to bacteria. By 2 dpe (7 dpf), the *E. coli* cell counts had increased nearly 10-fold to 7.8 ± 2.2 × 10^5^ CFU/larva, and by 4 dpe, it reached 3.9 ± 0.8 × 10^6^ CFU/larva (Fig. 4B). The sustained expression of fluorescent proteins over time suggested ongoing metabolic activity within the bacteria. Starting at 2 dpe, there was a noticeable change in bacterial colonization within the larvae gut. Rather than being uniformly distributed, bacterial cells began to concentrate in the digestive bulb and midportions of the larvae gut (Fig. S1; Fig. 4A), consistent with *E. coli* preferring specific sites within the gut (55, 56). Again, the LRE region exhibited sustained fluorescence during the experiment (50). When quantified, fluorescence increased over time but was not significantly different between the three time points (245.7 ± 51.2 AU for 0 dpe, 320.4 ± 90.1 AU for 2 dpe, and 459.9 ± 93.7 AU for 4 dpe). It is possible that the clustering of bacteria into some areas of the gut increases fluorescence to a point of saturation. The experiment was conducted with three groups of larvae to assess the reproducibility of the protocol. Every group represents five larvae from a different hatching (1 per week). We observed no significant difference between the three groups at 0 dpe and at 4 dpe (Fig. 4). We only observed a difference in a group on the second day after exposure, which we attributed to biological variability. Our protocol allows for a comparable initial bacterial intake (between 10⁵ and 10⁶), consistent with observations from other protocols (4–6, 25, 57, 58), while maintaining stability and resulting in increasing bacterial populations over 4 days (16, 24, 25, 58).

**Fig 5:**
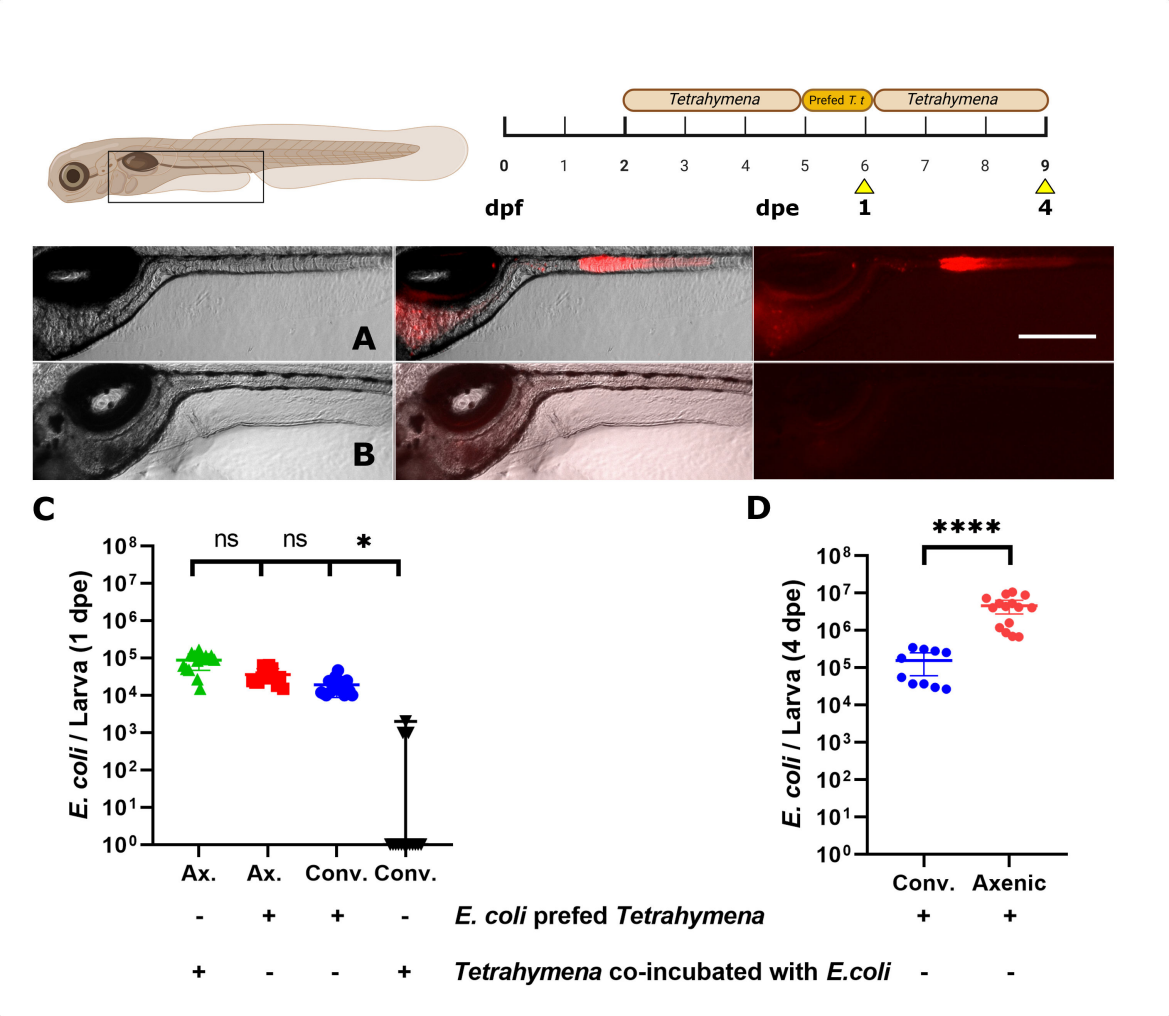
Colonization of conventionally raised larvae. (**A**) Lateral view of a conventionally raised zebrafish larvae after the colonization protocol with prefed *Tetrahymena* at 0 dpe. (**B**) Lateral view of a conventionally raised zebrafish larvae exposed to sterile *Tetrahymena.* (**C**) Concentration of *E. coli* expressing mCherry2 per larva at 1 dpe in conventional and axenic larvae, with or without pre-feeding of *Tetrahymena* with bacteria before addition to the media. (**D**) Concentration of *E. coli* expressing mCherry2 in axenic and conventionally raised larvae after 4 dpe. Scale bar: 300 µm. *N* = between 10 and 15. Statistical tests: Kruskal-Wallis and Dunn’s multiple comparison test for panel C (*P* value: * = 0.025, ns = 0.43 and 0.29) and Mann-Whitney (**** = *P* < 0.0001) for panel **D**.

While our results strongly suggest that *E. coli* colonization in the larval zebrafish gut was facilitated by *T. thermophila,* they did not confirm that the colonization occurs via the process described above. Thus, we next asked whether pre-feeding *Tetrahymena* with *E. coli*, allowing them to fill their vacuole with live bacteria, and then feeding the larvae with these bacteria-filled ciliates in bacteria-free media would result in a similar degree of colonization. This approach led to successful colonization of axenic zebrafish gut, to the same level as when bacteria and *Tetrahymena* were co-incubated in the water, consistent with the interpretation that *Tetrahymena* is an effective vector helping bacterial colonization (Fig. 5C, compare red and green dots).

We next aimed to ascertain whether *E. coli* could colonize the gut of zebrafish larvae possessing already a microbiota. Co-incubating bacteria and *Tetrahymena* in the media did not lead to significant bacterial intake in conventional larvae (Fig. 5C, black dots), whereas pre-feeding *Tetrahymena* with *E. coli*, as done above for axenic larvae, allowed *E. coli* colonization in the gut of conventionally raised zebrafish to the same extend as in axenic larvae (Fig. 5, compare blue and red dots).

In conventionally raised larvae, no antibiotics were used, unlike for axenic larvae, where antibiotics were applied for selective pressure. Despite the absence of ampicillin, bacterial fluorescence persisted in these conventionally raised zebrafish larvae for 4 dpe (Fig. 5D), suggesting that the plasmid was maintained in *E. coli* cells. The fluorescent *E. coli* cell counts were significantly lower in conventionally raised zebrafish larvae (1.6 ± 0.4 × 10^5^ CFU/larva) compared to axenic conditions (3.9 ± 0.8 × 10^6^) (Fig. 5D). The lower concentration of *E. coli* in conventionally raised zebrafish larvae gut is likely due to the presence of other competing bacterial populations, a phenomenon known as microbiota colonization resistance (59). Nonetheless, the protocol allowed colonization within the natural zebrafish larvae gut microbiota until at least 9 dpf. Longer observation periods would be required to determine whether the bacteria persist in the gut throughout later developmental stages, as changes in gut conditions may cause some bacteria to lose their ability to colonize. Although all experiments were performed in triplicate using groups of five larvae, some minor variability was observed. For example, in one group of five larvae, the bacterial concentration measured 5 days after exposure had a mean value of 6.4 ± 1.1 × 10^6^ CFU/larva.

### Colonization in different zebrafish transgenic lines

**Fig 6:**
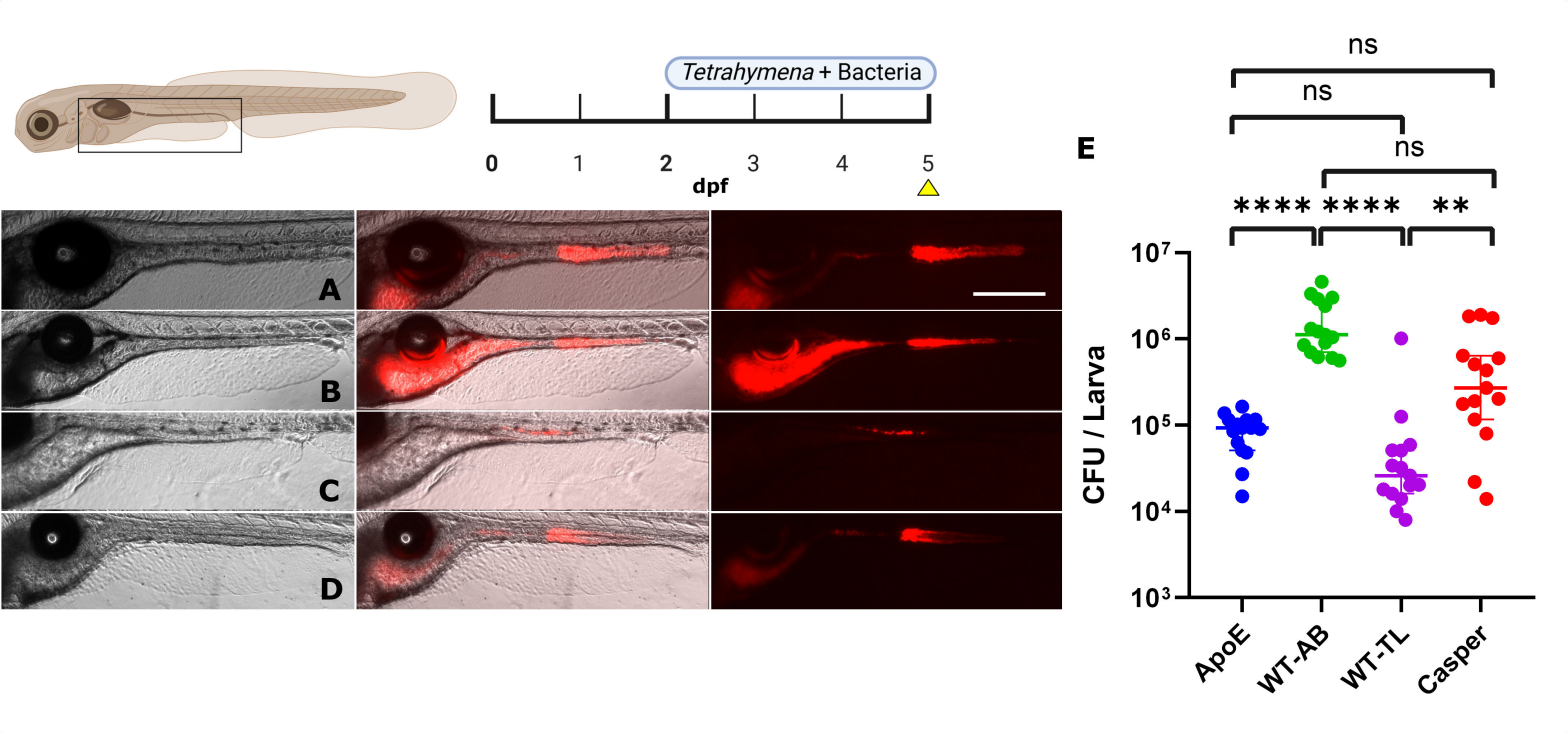
Colonization of four lineages of zebrafish larvae (5 dpf) with *E. coli* expressing mCherry2. (**A**) ApoE lineage. (**B**) WT-AB lineage. (**C**) WT-TL lineage. (**D**) CASPER lineage. (**E**) level of colonization per lineage. Scale bar: 300 µm. *N* = 15 for each condition. Statistical tests: a Kruskal-Wallis and Dunn’s multiple comparison test were performed, where ** denotes a significant difference (*P* = 0.0042), and **** indicates a highly significant difference (*P* < 0.0001) while ns is non-significative (*P* > 0.1).

The genetic diversity of zebrafish lines, along with the introduction of transgenes, may impact the gut-microbiota interactions, including the efficiency of bacterial colonization. It is well-established that both the genetics and the immune system of the zebrafish directly influence the composition of their gut microbiota (60–62). For example, the Ret tyrosine kinase, which is associated with gut movement, can influence gut colonization (63, 64). To assess the efficacy of our colonization protocol across various genetic zebrafish lines, we conducted a comparison involving two wild-type lines (WT-TL, WT-AB), a transgenic line tg(ApoEb:lyn-EGFP), and a pigmentless mutant line (Casper). The colonization was performed with the optimized protocol described above (2 to 5 dpf) on axenic larval zebrafish and quantified at 5 dpf. While all four zebrafish lines were successfully colonized, we observed different concentrations of bacteria in the gut among them. WT-AB had the highest CFU/larva (1.7 ± 0.3 × 10^6^), followed by Casper (5.8 ± 1.7 × 10^5^), WT-TL (9.9 ± 6.5 × 10^4^), and ApoE (8.7 ± 1.0 × 10^4^) (Fig. 6). Thus, the proposed protocol can lead to bacterial colonization in several zebrafish lines, albeit with different efficiency. Depending on the required zebrafish line under study, the colonization protocol may need adjustments.

### Multicolor bacterial colonization

**Fig 7:**
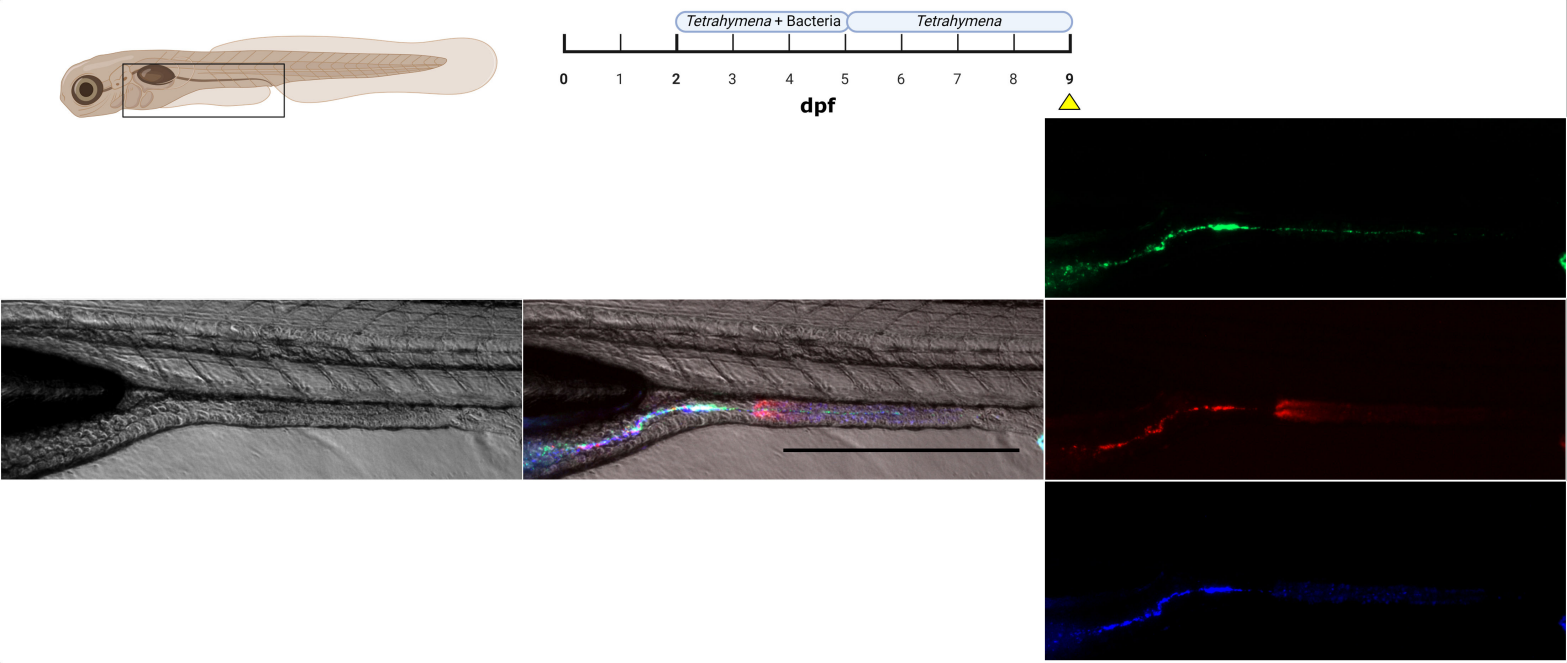
Multi-color bacterial colonization in larval zebrafish gut. Lateral view of a zebrafish (9 dpf/4 dpe) colonized with three strains of *E. coli,* each expressing a different fluorescent protein (top: *mNeonGreen*, middle: *mCherry2,* and bottom: *mCerulean3*). Scale bar: 600 µm.

The use of fluorescence to label bacteria offers the ability to discriminate between distinct microbial populations. To test for this possibility, we used three derivatives of the same *E. coli* strain, each expressing a different fluorescent protein (mCerulean3, mNeonGreen, mCherry2). We were able to follow with microscopy the colonization of three *E. coli* derivatives over 4 dpe (Fig. 7). Colonization was monitored over 1 week and showed similar timing and spatial patterns for colonization with a single fluorescent bacterial strain. When carrying the same plasmid backbone, the three *E. coli* strains displayed comparable colonization dynamics and growth kinetics. All imaging channels were evaluated for spectral bleed-through. mCherry2, mNeonGreen, and mCerulean3 have excitation/emission maxima of 589/610, 506/517, and 433/475 nm, respectively. Although these fluorophores have distinct spectral peaks, emission from mNeonGreen partially bleeds into the mCerulean3 detection channel, at approximately half the intensity. In addition, excitation of mNeonGreen can weakly excite mCerulean3 (∼2% of its maximal excitation; SpectraViewer, FPbase). This bleed-through was corrected by spectral unmixing, which enabled clear separation of signals originating from the mCerulean3 and mNeonGreen populations.

### Colonization with other bacteria

**Fig 8:**
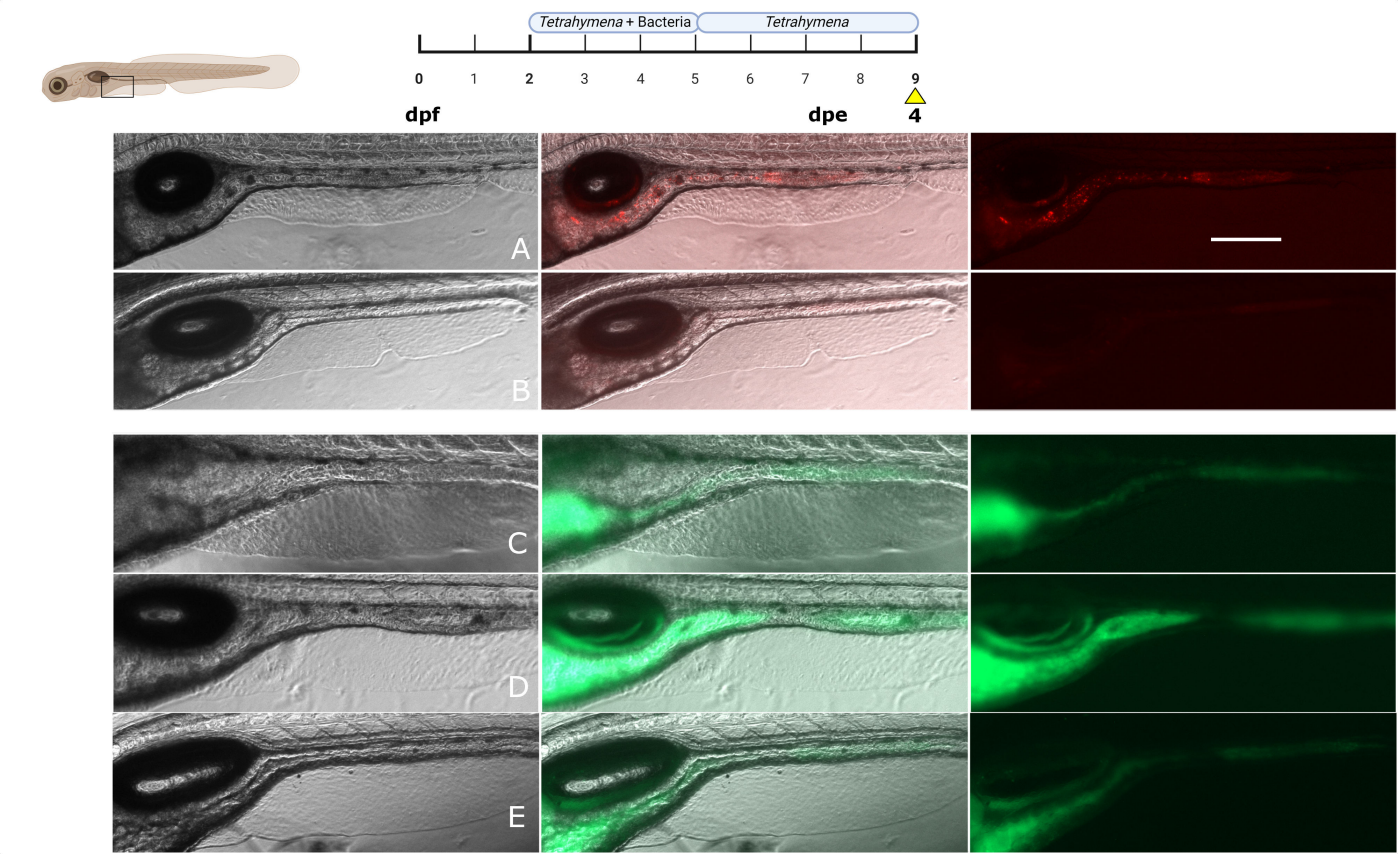
Colonization of larval zebrafish gut with endogenous bacterial strains. WT-TL axenic zebrafish larvae colonized with *A. veronii* expressing *mCherry2* (**A**), without bacteria (CTRL for red fluorescence) (**B**)*,* colonized with *V. cholerae* (**C**), and *P. aeruginosa* (**D**) expressing mNeonGreen, and without bacteria (CTRL for green fluorescence) (**E**). Larvae were imaged at 4 dpe. Autofluorescence from outside the gut can be observed in non-colonized larvae (**B–E**). Scale bar: 300 µm.

**Fig 9:**
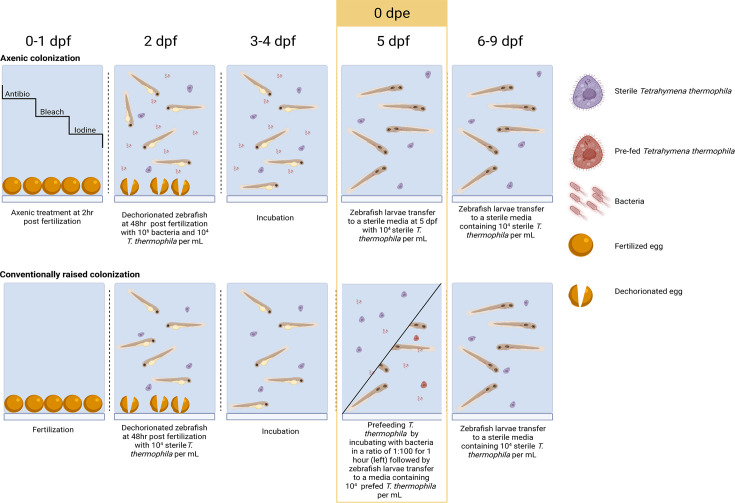
Schematic of the colonization protocols for axenic and conventionally raised zebrafish larvae.

To demonstrate the applicability of the optimized colonization protocol with other bacteria, we tested colonization of axenic zebrafish larvae with strains of *A. veronii*, *P. aeruginosa,* or *V. cholerae* expressing mCherry2 or mNeonGreen. These bacterial species are typically prevalent in the zebrafish gut at 5 dpf (16, 28). Distributed fluorescence clusters were observed for 4 dpe in the mid-gut section of the larvae colonized with *P. aeruginosa* (Fig. 8D). Bacterial plating also showed the presence of those bacteria at 4 dpe. For *A. veronii* and *V. cholerae*, a lower fluorescent signal was observed in the mid-gut (Fig. 8A and C), which may be caused by the absence of a synergy with other bacteria needed for better colonization, or they may be less adapted to this environment. In the case of *A. veronii*, *V. cholerae,* and *P. aeruginosa*, little is known about synergistic interactions in zebrafish. To our knowledge, the only bacterial pair that has been studied is *Vibrio–Aeromonas (*64). Co-colonization of these two bacteria in zebrafish has been shown to result in competition between the populations as the *Aeromonas* population decreases when *Vibrio* is present. This shift in population dynamics was attributed to the challenge posed by *V. cholerae* and to gut motility, as *Aeromonas* is less adapted to the mechanical movement of the zebrafish intestine. It would therefore be interesting to investigate the interactions of *P. aeruginosa* with other bacterial species. Nonetheless, the above results indicated colonization by other bacterial species that are commonly found in the gut of zebrafish, showing the effectiveness of the selected protocol with the tested parameters (Fig. 9).

### Conclusion

In this study, we have tested different parameters to optimize bacterial colonization and growth in the gut of the zebrafish larvae. By timing bacterial exposure at hatching, we ensured early colonization, mitigating the effects of a germ-free environment. Furthermore, placing multiple larvae in a single well demonstrates that the method can be scaled to study larger groups of fish while also enabling biological replication. Incorporating *Tetrahymena thermophila* was shown to enhance bacterial colonization of the gut. Fluorescence in the gut and cell counts confirmed bacterial viability throughout the incubation period, enabling longitudinal studies. Tested over a 9-day period, covering a crucial early developmental phase of zebrafish, this protocol opens the door to introducing a synthetic microbiota composed of multiple strains. Monitoring these strains via fluorescence could shed light on their impact on fish development and health, particularly by studying the crosstalk between the gut microbiota and its host. However, because our observations were limited to 9 dpf, it remains to be seen if this approach leads to lifelong colonization in juvenile and adult zebrafish.

## SUPPLEMENTAL MATERIAL

Figure S1 spectrum.02853-25-s0001.docx The areas of the zebrafish larvae gut at the age of 9 dpf.

Movie S1 spectrum.02853-25-s0002.avi Swimming *Tetrahymena* engorged with *E. coli* expressing mNeonGreen. Bacteria can stay alive during multiple hours in the ciliate, making it a suitable vector for colonization.

Movie S2 spectrum.02853-25-s0003.avi Imaging of 7 dpf zebrafish larvae colonized with red fluorescent *E. coli* over 8 hours every 15 minutes.

ASM does not own the copyrights to Supplemental Material that may be linked to, or accessed through, an article. The authors have granted ASM a non-exclusive, world-wide license to publish the Supplemental Material files. Please contact the corresponding author directly for reuse.
