# Supplementary material for: Optimization of bacterial colonization in the gut of axenic and conventional zebrafish larvae using live food
Source: Microbiol Spectr. 2026 Apr 21;14(6):e02853-25. doi: 10.1128/spectrum.02853-25 (PMC13228016; doi:10.1128/spectrum.02853-25)
Supplement: Figure S1 — The areas of the zebrafish larvae gut at the age of 9 dpf. [file spectrum.02853-25-s0001.docx]

**Movie 1:** Swimming *Tetrahymena* engorged with *E. coli* expressing mNeonGreen. Bacteria can stay alive during multiple hours in the ciliate, making it a suitable vector for colonization. ^20,62^

**Movie 2:** Imaging of 7 dpf zebrafish larvae colonized with red fluorescent *E. coli* over 8 hours every 15 minutes. 1 frame = 15 minutes.


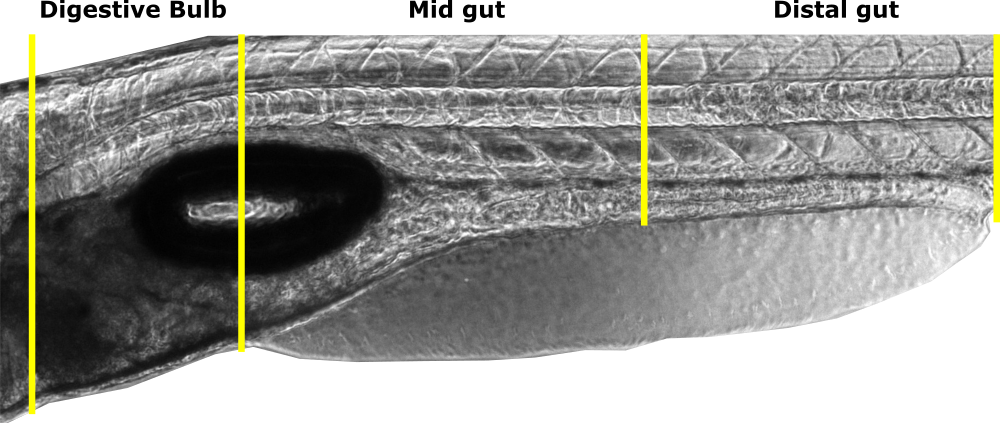


Supplementary Figure 1: The areas of the zebrafish larvae gut at the age of 9 dpf.
